# Pediatric Gastric Adenocarcinoma Presenting As Hip Pain

**DOI:** 10.7759/cureus.34651

**Published:** 2023-02-05

**Authors:** Mohammed J Sobh, Obada A Al Jayyousi, Ahmad Mahasna, Rawan J Sobh

**Affiliations:** 1 General Practice, King Abdullah University Hospital/Jordan University of Science and Technology, Ar Ramtha, JOR; 2 Surgical Oncology, Jordanian Royal Medical Services, Amman, JOR; 3 Pediatrics, Mubarak Al-Kabeer Hospital, Jabriya, KWT

**Keywords:** rare cancer, rare case, metastasis, gastric adenocarcinoma, pediatric tumor

## Abstract

The third most lethal cancer in the world is gastric adenocarcinoma, which is uncommon in children. Patients with gastric adenocarcinoma typically experience vomiting, abdominal pain, anemia, and weight loss. We present a case of a 14.5-year-old male with gastric adenocarcinoma that manifested as left hip pain, epigastric pain, dysphagia, weight loss, and melena. Physical exam revealed cachexia, jaundice, a palpable epigastric mass, palpable liver edge, and left hip tenderness.

Laboratory tests showed microcytic anemia, increase in carcinoembryonic antigen (CEA), and abnormal liver function test. Endoscopy revealed a cardial mass extending to the esophagus involving the gastroesophageal junction (GEJ). The gastric mass biopsy was consistent with invasive, moderately-differentiated gastric adenocarcinoma, which confirmed the diagnosis of gastric adenocarcinoma. Furthermore, a bone isotope scan revealed mildly hypervascular active bone pathology within the left proximal femur implying possible metastasis. Computed tomography scans and barium swallow were also helpful in supporting the diagnosis. Our case report emphasizes that gastric adenocarcinoma should be encompassed in the differential diagnosis of pediatric patients with hip pain.

## Introduction

Gastric adenocarcinoma is a rare diagnosis in children but it is the third deadliest cancer worldwide [1]. It constitutes 0.05% of all gastrointestinal cancers in childhood, and it accounts for only 5% of all pediatric neoplasms [2,3]. Patients with gastric adenocarcinoma commonly present with complaints of vomiting, abdominal pain, anemia, and weight loss [1]. The prognosis is poor in patients with metastasis at the time of diagnosis, with a mean survival of approximately three months [1]. The exact cause of stomach cancer in children is still unknown, despite the knowledge that several environmental and genetic variables, in particular nutrition and lifestyle, are risk factors in adults [4]. Here, we present a very uncommon case of pediatric stomach cancer.

## Case presentation

A 14.5-year-old male patient presented to the outpatient clinic with left hip pain for three months. The pain was dull, achy, with sudden onset, and non-radiating. Moreover, his pain was not relieved by non-steroidal anti-inflammatory drugs (NSAIDs) and was associated with fatigue and dizziness. He also reported mild, intermittent epigastric pain of gradual onset, associated with dark, tarry stools and significant weight loss (60 kg to 40 kg) over the last two months. These symptoms prompted the patient's family to seek medical attention, and blood units were given accordingly. However, the patient was discharged against medical advice before any further investigations. Starting a month ago, the patient also developed dysphagia and loss of appetite. All of which justified admitting the patient for further investigations. It’s worth mentioning that there was no family history of gastric cancer or any other type of cancer, and his parents were not consanguineous. No other significant history or medications were noted, and the review of systems was otherwise unremarkable.

On physical examination, the patient looked cachectic and in pain. Temporal wasting and jaundice over the sclera were also evident. Upon palpation, left hip tenderness, a palpable epigastric mass, and a palpable liver edge were confirmed. Furthermore, the left supraclavicular lymph node was not enlarged (Virchow's node). Cell blood count (CBC) and red blood cell (RBC) indices were consistent with microcytic anemia (Table 1). Labs also revealed an increase in carcinoembryonic antigen (CEA) (Table 2), liver enzymes, total bilirubin, and alkaline phosphate (ALP) (Table 1).

**Table 1:** Laboratory investigations RBC: red blood cell; HB: hemoglobin; HT: hematocrit; MCV: mean corpuscular volume; MCH: mean corpuscular hemoglobin; MCHC: mean corpuscular haemoglobin concentration; RDW: red cell distribution width; WBC: white blood cells; PLT: platelet; ALT: alanine aminotransferase; AST: aspartate aminotransferase; PT: prothrombin time; INR: international normalized ratio; ALP: alkaline phosphatase; HCG: human chorionic gonadotropin; AFP: alpha fetoprotein

Test	Value	Reference range
RBC	2.99	4.1-5.5 million/mcl
HB	5.8	13.3-16.9 g/dL
HCT	19.6	38- 47%
MCV	65.5	82.5-98 fL
MCH	19.4	25- 30 pg
MCHC	29.6	31-34) g/dL
RDW	18	11.4- 13.5%
WBC	13.9	(3.8- 10.4) 10^3^/uL
PLT	501	(139- 320) 10^3^/uL
Bilirubin Total	1.68	0.1-1.2 mg/dL
ALT	37	<=41 U/L
AST	98	<=37 U/L
PT	17.7	12.7-16.1 sec
INR	1.33	0.8-1.2
ALP	801	74-390 U/L
B-HCG	0.378	As tumor marker: ND -2.5 mIU/ml
AFP	4.44	0.0-40 ng/mL

**Table 2:** Carcinoembryonic antigen tumor marker CEA: carcinoembryonic antigen

Tumor Marker	At admission	Two weeks after	Three weeks after	Four weeks after	Reference range
CEA ng/ml	100	876	992.6	1000	Up to 3 in non-smoker

Endoscopy was performed that showed a circumferential, fragile, ulcerating cardial mass extending to the esophagus involving the gastroesophageal junction (GEJ) and gastric polyps, from which biopsies were taken for histopathology examination. The gastric mass biopsy was consistent with invasive, moderately differentiated gastric adenocarcinoma and the biopsy taken from the gastric mucosa was positive for *Helicobacter pylori*. Additionally, the patient underwent chest, abdomen, and pelvis CT with IV contrast, Tc 99m-methylene diphosphonate (MDP) bone isotope scan (3 phase + whole body (WB)), and barium swallow.

**Figure 1:**
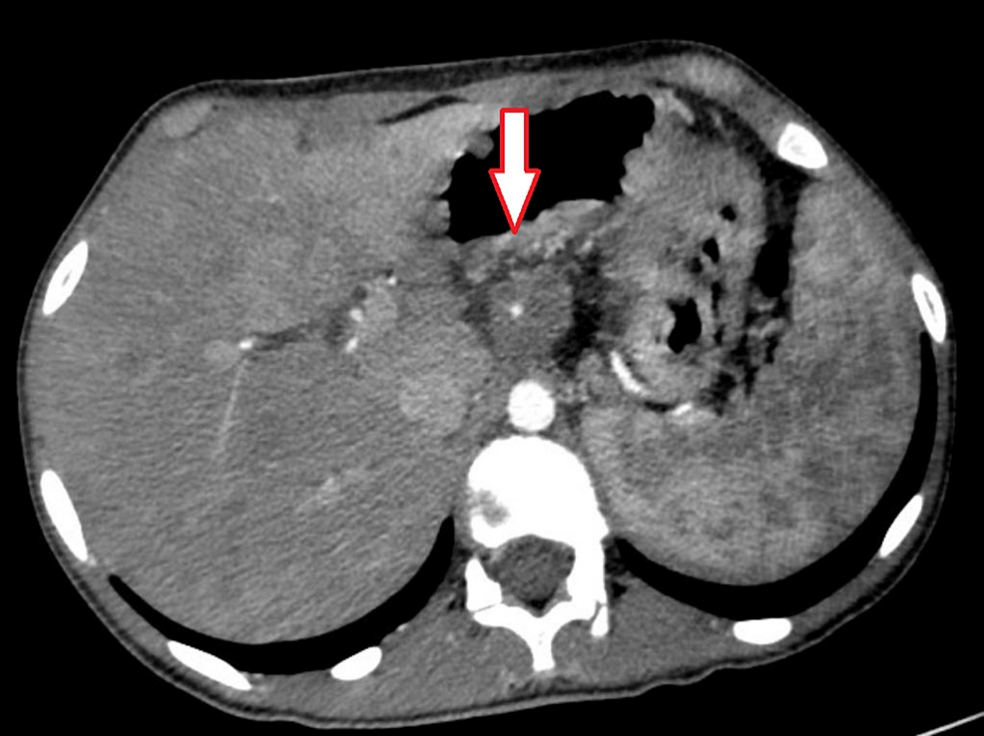
CT abdominal scan revealing GEJ thickening, supporting the presence of a mass GEJ: gastroesophageal junction

**Figure 2:**
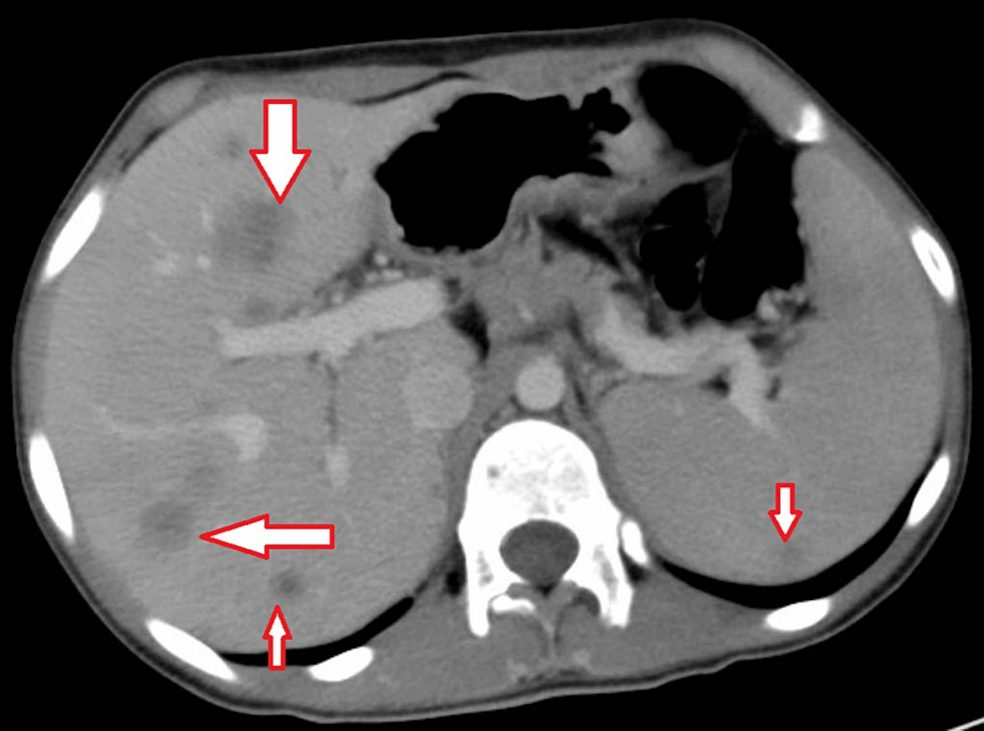
CT abdominal scan showing the presence of multiple liver lesions and a possible splenic lesion, which could reflect metastasis

**Figure 3:**
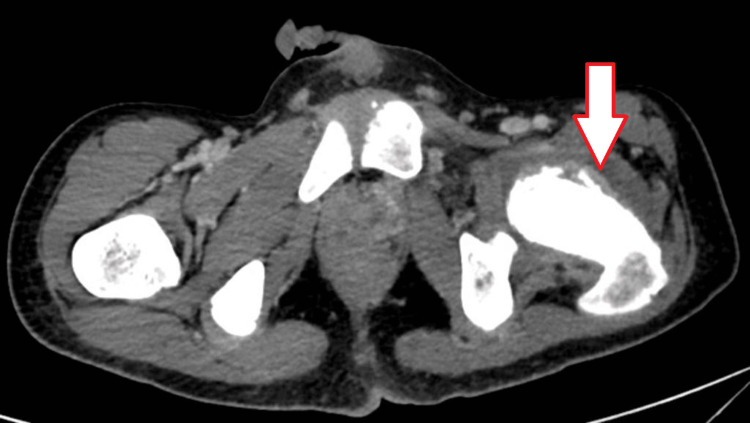
CT pelvic scan showing the presence of a suspicious lesion involving the left femoral neck

To check for the extent of tumor spread and metastasis, a chest, abdomen, and pelvis CT was performed that revealed a suspicious thickening of the GEJ and the lesser curvature of the stomach (Figure 1) along with multiple liver and possible splenic metastasis (Figure 2). A highly suspicious lesion involving the left femoral neck was also seen (Figure 3). 

**Figure 4:**
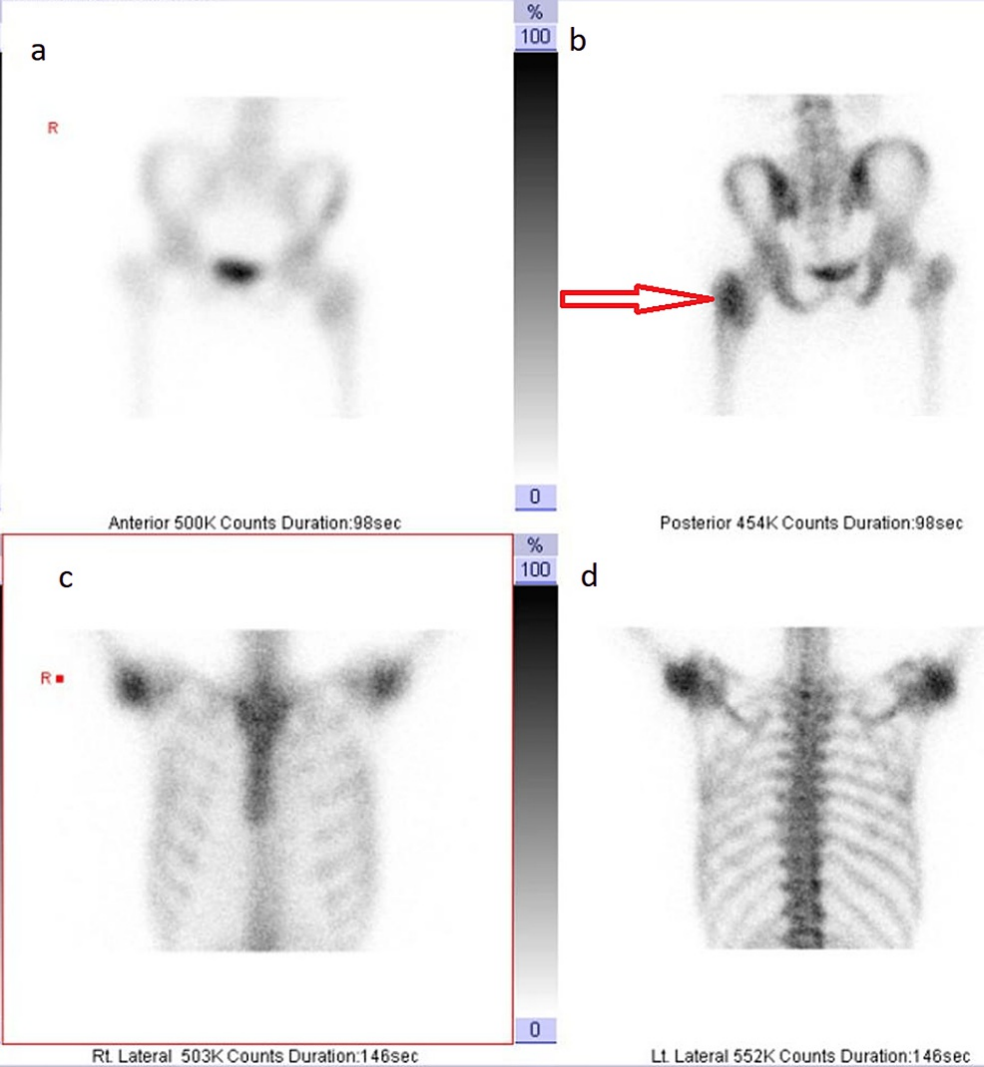
Bone isotope scan showing the amounts of radioactive substances absorbed, ranging from (0-100) (b) A mildly hypervascular, active bone pathology within the left proximal femur is noted; (a, c, d) There is no evidence of any other suspicious bony lesions

To further assess the lesion involving the left femoral neck, a bone isotope scan was performed. It showed a mildly hypervascular active bone pathology within the left proximal femur (Figure 4b) with no evidence of any other suspicious bony lesions (Figures 4a, 4c, 4d).

**Figure 5:**
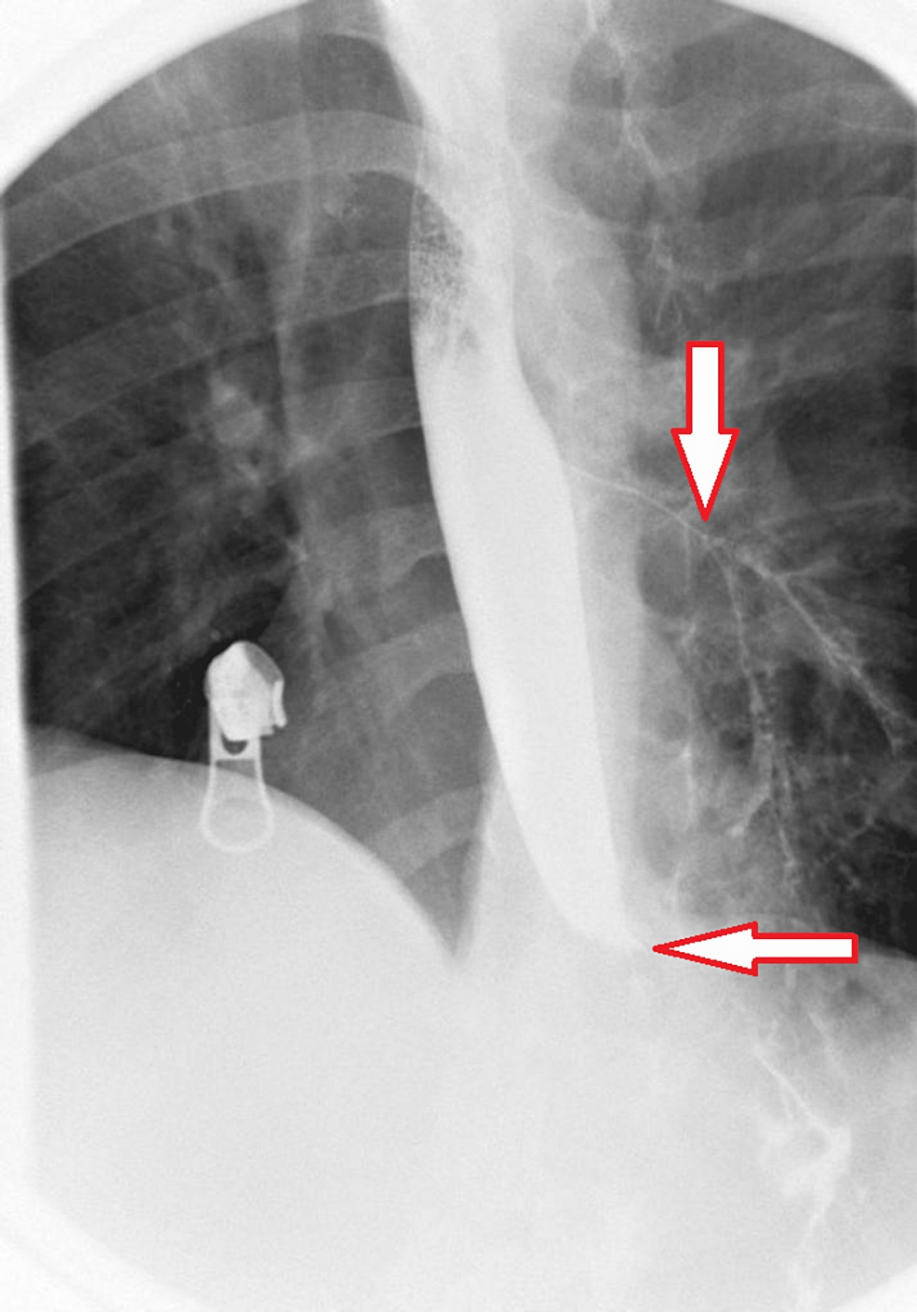
Barium swallow showed significant hold-up of contrast at the distal end of the esophagus and impaired swallowing mechanism with aspiration of contrast

Dysphagia and the extent of obstruction resulting from the mass were evaluated by a barium meal. Significant hold-up of contrast at the distal end of the esophagus and impaired swallowing mechanism with aspiration of contrast were noted (Figure 5).

Management

Since oral feeding was insufficient, we tried a nasogastric tube at first, but failed. So, jejunostomy was done, with the placement of a feeding tube. Pain was mainly managed with morphine and followed by the palliative team. The patient was eventually scheduled for chemotherapy; however, he passed away of cardiovascular collapse before receiving any of it, 16 days after admission.

## Discussion

Gastric cancer is a challenging diagnosis in children and is considered rare in children with very limited information on the clinical presentation and outcome [4]. Pediatric gastric adenocarcinomas are not considered primarily in differential diagnosis because they are very rare. It is emphasized that the etiology in patients with adult gastric adenocarcinoma is multifactorial due to lifestyle, dietary, and infectious factors. However, the reason for the development of gastric adenocarcinoma in children and the mechanism is still unclear [4].

In a study conducted using the United States National Cancer Database, 0.1% of gastric adenocarcinoma cases were pediatric cancer and pediatric cases were more advanced and poorly differentiated than those of adults. It was also stated that young adults were appreciably more likely to present with bone metastases compared to older patients [5].

It’s also worth mentioning, that gastric adenocarcinoma at the cardia, as in our case, tends to have a poorer prognosis when compared to other sites [6]. To elaborate, a study in adults has shown that gastric adenocarcinoma at the cardia has a higher incidence of lymph node metastasis and invasion of serosa, blood vessels, and lymphatics than other sites of the stomach [6]. Results of that study have also revealed a lower five-year survival rate of gastric adenocarcinoma at the cardia in comparison to other sites.

The chance of long-term survival in patients with gastric adenocarcinoma is only possible with total surgical resection of the tumor, but patients are not always candidates for surgery because of their widespread metastases during diagnosis [1]. Young people at risk of hereditary diffuse gastric cancer may benefit, however, from prophylactic gastrectomy [7]. Although the results of some studies were consistent with an inversely proportional relation between *H. Pylori *and gastric adenocarcinoma at the cardia, the patient in our case was positive for *H. Pylori* [8].

A genetic test was not performed for our patient. However, there are genetic associations that have been reported for pediatric gastric adenocarcinoma. In one study, gastric adenocarcinoma was present in an atypical case of Li Fraumeni syndrome [9]. In another study, it has been seen in association with IPEX (immune dysregulation, polyendocrinopathy, enteropathy, X-linked) syndrome [10]. There is also evidence that germ-line truncating mutations in the E-Cadherin (CDH1) gene are associated with hereditary, diffuse gastric cancer. Therefore, asymptomatic carriers of these types of mutations are advised to get screened and consider prophylactic gastrectomy, especially with a positive family history of diffuse gastric cancer [7].

Endoscopy of our patient has shown gastric polys, to which there might be a link to gastric adenocarcinoma, as supported by the literature [11]. There is a rare polyposis syndrome, defined by the presence of proximal gastric polyps and gastric adenocarcinoma. It has an autosomal dominant mode of inheritance, and thus family members at risk should be screened for the associated genetic mutations [11].

There was a marked elevation of CEA in the course of the disease of the patient in our case. On the other hand, other tumor markers could be elevated. A study by Emir and colleagues reported a mild elevation of alpha-feto protein levels in a 12-year-old boy with gastric adenocarcinoma of hepatoid origin [12].

Our case presented with non-specific symptoms such as hip pain, with multiple metastases at the time of diagnosis. Interestingly, there is a case reported with a similar presentation [1]. There have been other presentations reported for pediatric gastric cancer including ascites and gastric outlet obstruction [13,14]. Due to these case reports, it is emphasized that gastric adenocarcinoma should be included in the differential diagnosis of hip pain in pediatric patients. This is of special significance in developing countries where low education and socio-economic status are not uncommon, possibly explaining the not following through with initial gastrointestinal complaints. In the diagnosis of the disease, upper GI endoscopy and histopathological examination of endoscopic biopsies were very useful for diagnosis. Clinical preliminary information about the disease was obtained by radiological imaging methods.

## Conclusions

Gastric adenocarcinoma is not common in the pediatric age group. Children with this diagnosis often present with vomiting, abdominal pain, anemia, and weight loss. This is a rare case of a child with pediatric gastric adenocarcinoma, presenting with a chief complaint of hip pain, along with other symptoms. Our case adds another presentation with a unique finding to the literature, supporting the growing differential diagnosis of gastric adenocarcinoma for children presenting with hip pain, especially in developing countries with low awareness.
